# Smoking Prevalence among Migrants in the US Compared to the US-Born and the Population in Countries of Origin

**DOI:** 10.1371/journal.pone.0058654

**Published:** 2013-03-08

**Authors:** Jizzo R. Bosdriesz, Nienke Lichthart, Margot I. Witvliet, Wim B. Busschers, Karien Stronks, Anton E. Kunst

**Affiliations:** Department of Public Health, Academic Medical Centre, University of Amsterdam, Amsterdam, The Netherlands; The University of Auckland, New Zealand

## Abstract

**Objectives:**

Smoking among migrants is known to differ from the host population, but migrants’ smoking is rarely ever compared to the prevalence of smoking in their country of origin. The goal of this study is to compare the smoking prevalence among migrants to that of both the US-born population and the countries of origin. Further analyses assess the influence of sex, age at time of entry to the US and education level.

**Methods:**

Data of 248,726 US-born and migrants from 14 countries were obtained from the Tobacco Use Supplement to the Current Population Survey (TUS-CPS) from 2006–2007. Data on 108,653 respondents from the corresponding countries of origin were taken from the World Health Survey (WHS) from 2002–2005.

**Results:**

The prevalence of smoking among migrants (men: 14.2%, women: 4.1%) was lower than both the US-born group (men: 21.4%, women: 18.1%) and countries of origin (men: 39.4%, women: 11.0%). The gender gap among migrants was smaller than in the countries of origin. Age at time of entry to the US was not related to smoking prevalence for migrants. The risk of smoking for high-educated migrants was closer to their US counterparts.

**Conclusions:**

The smoking prevalence among migrants is consistently lower than both the country of origin levels and the US level. The theory of segmented assimilation is supported by some results of this study, but not all. Other mechanisms that might influence the smoking prevalence among migrants are the ‘healthy migrant effect’ or the stage of the smoking epidemic at the time of migration.

## Introduction

Smoking among migrants has been a topic of growing scientific interest in the past decades, especially in the United States (US). A common finding is that migrants in general and especially female migrants smoke less than the US-born population. [1] This strongly affects differences in health between migrants and the host population. One study found that the life expectancy of both foreign- and native-born Hispanics was higher than for US-born Whites, and that more than 50% of this difference was attributable to smoking. [2].

The differences in smoking between migrants and the host population can be attributed to a number of factors, including social norms, gender role equality and acculturation. [3], [4] Acculturation is the process of psychological and cultural adaptation that takes place when two cultures meet, as happens in migration. [5] This theory however, is not without criticism; the theory of segmented assimilation might be a better alternative. [6], [7] Segmented assimilation states that although migrants do take up some values and behaviours of the host culture, they also maintain a strong ethnic identity. It is likely that norms regarding public or functional topics are readily adapted while private or social values are more persistent. [8].

Research on smoking among migrants in the US to date has two main limitations: first, most studies focus on Mexican and Hispanic migrants, [1], [9] or Asian migrants. [3] Second, while these studies compared the migrant groups to the US population, they did not make comparisons to the population in the countries of origin. Therefore the influence of the smoking prevalence in the country of origin on migrants’ smoking is unknown.

The main objective of this study is to compare the smoking prevalence among migrants to that of the US-born population as well as the population of the country of origin. Data on migrants to the US from 14 countries across all continents will be compared to the US-born population and to the population of their respective countries of origin. The structure of this study is based on four specific hypotheses:

1. Based on the theory of segmented assimilation, we expect that the norms and values of the countries of origin still influence the behaviour of migrants. Therefore we expect that the smoking prevalence among migrants shows a pattern similar to the prevalence in the countries of origin. More specifically, migrants from a country with an especially low smoking prevalence are expected to smoke less than other migrant groups.
2. Earlier studies have shown that in developing countries the gap in smoking prevalence between men and women is still very large, while in developed countries like the US it has decreased to a large extent. [4] Other research shows that more assimilated men smoke less, while more assimilated women smoke more, thereby decreasing the gender gap. [1], [3], [9] We therefore expect that the ‘gender gap’ in smoking prevalence will be smaller among migrants than in their countries of origin, but still larger than among the US-born.
3. It is often seen that second generation migrants or those who migrated at a young age are more successful in terms of educational and economic development. [7] We hypothesize that migrants who migrated at a younger age and second generation migrants will be more similar to the host population in their smoking prevalence than those who migrated later in life.
4. Like many behaviours, smoking often follows a socioeconomic gradient, although this may differ per country, depending on the stage of the tobacco epidemic. [10] In the process of migration, a higher educational status is associated with greater assimilation to the host culture. [5] Therefore we hypothesize that the smoking prevalence of higher educated migrants is similar to the high-educated US-born group, while the lower educated migrants’ smoking prevalence differs substantially from lower-educated US-born.

## Methods

### Tobacco Use Supplement to the Current Population Survey

Data on smoking of US residents were taken from the Tobacco Use Supplement to the Current Population Survey (TUS-CPS). [11] The TUS-CPS delivers nationally and state level representative data on smoking in the US household population. The survey has a civilian, non-institutionalized population of 15 years and older. The majority of the survey consists of self-reported measures, which were obtained by telephone for 70% of respondents and in person for the remaining 30%. The May 2006, August 2006 and January 2007 TUS-CPS were combined, as recommended by the Census Bureau, to maximize statistical power. [11] The TUS-CPS contains information of 287,991 respondents in the 2006/2007 survey period. The non-response rates were 19.3% for May 2006, 18.3% for August 2006 and 14.8% for January 2007. Weights were applied as instructed in the TUS-CPS technical documentation. [11].

The respondents of the TUS-CPS were asked about their own country of birth as well that of their parents. These answers were combined to select three groups for further analyses; US-born (US-born with US-born parents), migrants (foreign-born with foreign-born parents) and second generation migrants (US-born with foreign-born parents). An additional criterion for selection into the second and third group was that father and mother were born in the same country, and this country belongs to one of the 14 countries of origin (see below). A total of 40,832 respondents were not included in one of these groups, mainly because their parents were not born in the same country or due to missing data. This resulted in a final sample of 247,159 respondents (all 15+ years) who were included in the regression analyses. Further information on this population is shown in table 1. For comparing the prevalence of smoking among migrants and the US-born population, 9,500 respondents aged 15–17 were excluded to optimize comparability with the WHS.

**Table 1:** Descriptive information of the US study population by country of origin^1^.

		N			Education^2^			Age at entry to the US			
		Total	Male	Female	Low	Middle	High	2^nd^ Gen^3^	0–19	20–39	40–85
	**U.S.A**	225,981	107,087	118,894	32,956	115,755	77,270	–	–	–	–
**Africa**	**Ethiopia**	132	70	62	23	73	36	1	35	79	17
	**Other Africa** *	250	136	114	17	102	131	14	62	157	17
**The Americas**	**Brazil**	354	157	197	77	162	115	12	94	207	41
	**Dom. Rep.**	919	390	529	382	392	145	145	294	388	92
	**Ecuador**	451	231	220	137	205	109	54	147	212	38
	**Guatemala**	671	413	258	433	189	49	41	274	327	29
	**Mexico**	12,259	6,483	5,776	7,084	4,172	1,003	2,266	4,854	4,448	687
**Asia**	**China**	1,514	690	824	316	466	732	205	293	755	261
	**Laos**	221	104	117	84	103	34	54	83	68	16
	**Pakistan**	181	102	79	30	57	94	21	50	88	22
	**Philippines**	2,328	977	1,351	270	853	1,205	413	611	996	308
	**Vietnam**	987	463	524	252	442	293	132	310	379	166
**Europe**	**Russia**	668	292	376	95	249	324	238	125	188	117
	**Ukraine**	243	118	125	24	81	138	49	61	80	53
**Total**		247,159	117,713	129,446	42,180	123,301	81,678	3,645	7,293	8,372	1,864

1 Country of origin is country of birth, except for second generation migrants where it is the country of birth of the parents.

2 Education: Low = no higher than ‘12^th^ grade without diploma’, Middle = from ‘high school diploma or equivalent’ to ‘college but no degree’, High = from ‘associate degree’ to ‘doctorate degree’.

3 2^nd^ Generation: those born in the US whose parents are both foreign-born.

* Other Africa: Ghana, Kenya and South Africa.

### World Health Survey

Data on the populations of the countries of origin of the various migrant groups were obtained from the World Health Survey (WHS). [12] The WHS was developed by the World Health Organization (WHO) in order to gather comparable baseline information about the health of populations in 72 countries from across six continents. One research methodology was applied throughout all participating countries to improve cross-national comparability. More details can be found on the WHO website. [13] Participating countries were given a choice of three pretested methods: household face-to-face surveys, computer assisted telephone interview, and computer assisted personal interview. Sample sizes varied between 1,000 and 10,000 per country. Respondents were randomly selected and 18+.

Countries from the WHS were included in this study if data on at least 100 migrants from that country were included in the TUS-CPS. Data from 17 countries were considered:

Ethiopia, Ghana, Kenya and South Africa;

The Americas: Brazil, Dominican Republic, Ecuador, Guatemala and Mexico;

Asia: China, India, Laos, Pakistan, Philippines and Vietnam;

Russia and Ukraine.

The WHS sample of India was unrepresentative for India as a whole and therefore was not included in this study. In order to have large enough samples, three African countries (Ghana, Kenya and South Africa) were combined into ‘other Africa’ which was considered a single country from this point on. WHS data for the 14 selected countries of origin contained a total of 108,653 respondents.

### Ethics Statement

In the Netherlands, medical research is governed by the Medical Research Involving Human Subjects Act (WMO), which is based on the principles of the declaration of Helsinki. This law only applies if study participants are subjected to any action, treatment or behaviour. We have written confirmation from the Medical Ethics Review Committee of the AMC that the WMO does not apply to this study and therefore no official approval was required.

### Variables

To determine smoking status in the TUS-CPS, the respondents were classified as ‘current smoker’ if their smoking status was ‘everyday smoker’ or ‘some days smoker’. They were classified as ‘non-current smokers’ if their smoking status was ‘former smoker’ or ‘never smoker’.

The age at entry to the US was calculated from the age at the time of the survey and the year of entry to the US. Age at entry was categorized into 3 groups: 0–19, 20–39 and 40–85. The group ‘second generation migrants’ (as described above) was added as a fourth category.

In the TUS-CPS respondents were asked to state their highest completed education. Based on this, they were divided into 3 groups. The respondents who had completed no more than ‘12th grade with no diploma’, were placed into the group ‘Low’. The respondents who had anything between a ‘high school grade-diploma or equivalent’ and ‘some college but no degree’ were placed into the group ‘middle’. Respondents with either an ‘associate degree-occupational/vocational’ or ‘doctorate degree’ were placed into the group ‘high’.

In the WHS, respondents were asked ‘Do you currently smoke any tobacco products such as cigarettes, cigars or pipes?’ Those who answered ‘Daily’ or ‘Yes but not daily’ were considered ‘smoker’, those who answered ‘No, not at all’ were classified ‘non-smoker’.

### Statistical Analyses

From the data of the WHS, age-standardized smoking prevalence rates were calculated (in Stata version 11.1) for each of the 14 countries of origin by use of the direct method of standardization and the WHO’s world standard population. [14] Prevalence rates were calculated per country and also stratified by sex. The same method and the same standard population were applied to data from the TUS- CPS to calculate the prevalence of ‘current smokers’ among the US-born and the migrant groups.

In further analyses, migrants were compared to the US-born group, for each migrant group separately. We calculated the relative risk (RR) with a 95% confidence interval (95% CI) with the US born as the reference group. These RR’s were estimated by use of generalized linear models with a log link function and a binominal error distribution. Observations were weighted with respect to the survey design and sampling weights provided by TUS-CPS. [11].

The RR’s were tested for interaction between country of origin and sex, education and age at migration. Interaction terms were investigated for significance using the working likelihood ratio (Rao-Scott) test. Three additional analyses were then performed, with different stratifications. The first was stratified by sex, the second by education and the third by age at migration. All analyses on the data from the TUS-CPS were performed using R (version 2.13.1) with the package ‘Survey’.

## Results

**Figure 1:**
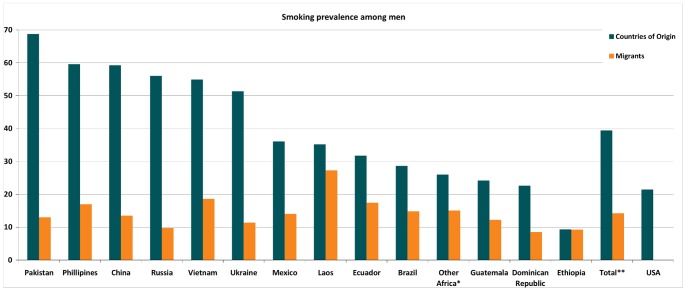
Prevalence of smoking for male migrants and their countries of origin compared to the US. *Other Africa: Ghana, Kenya and South Africa. ** Total does not include US-born.

**Figure 2:**
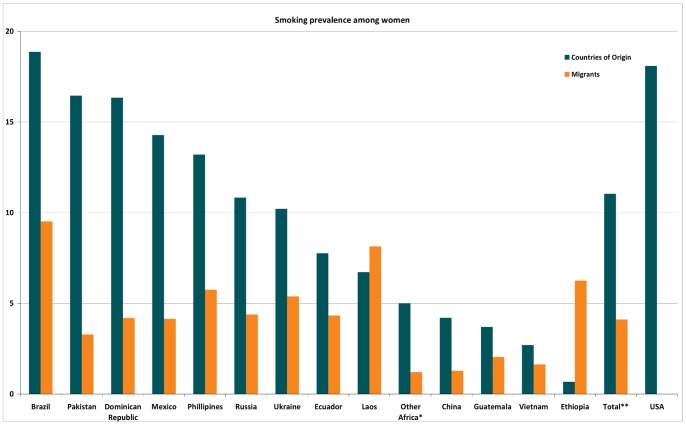
Prevalence of smoking for female migrants and their countries of origin compared to the US. *Other Africa: Ghana, Kenya and South Africa. **Total does not include US-born.

As shown in figure 1, the prevalence of smoking among all groups of male migrants, on average (14.2%) is lower than US-born men (21.4%) and in the combined group of all countries of origin (39.4%). Figure 2 shows that the prevalence of smoking among all female migrants (4.11%) is lower than among US-born women (18.1%) and their countries of origin (11.0%). In short, all migrants smoke less than both the US-born and their countries of origin’s populations. Some exceptions are observed among migrants from Brazil, Ethiopia and Laos.

**Figure 3:**
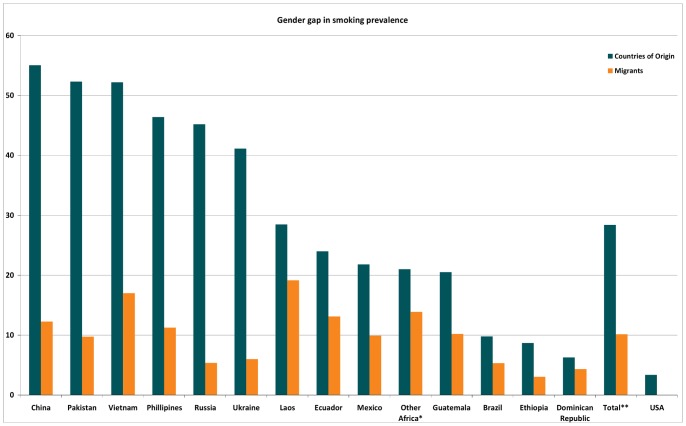
The smoking prevalence among migrants, compared to the smoking prevalence of their countries of origin and the US-born*. *USA refers to the US-born population, without migrants; therefore the y value is set at 0.

On the whole, the pattern of variations in smoking prevalence between the different migrant groups is not very similar to that in the countries of origin. Shown in figure 3, there is only a weak relationship between the smoking prevalence of migrants and that in their countries of origin. The dissimilarity in patterns is mainly due to the fact that migrants from Asia and Eastern-Europe have a much lower smoking prevalence than their countries of origin.

**Figure 4:**
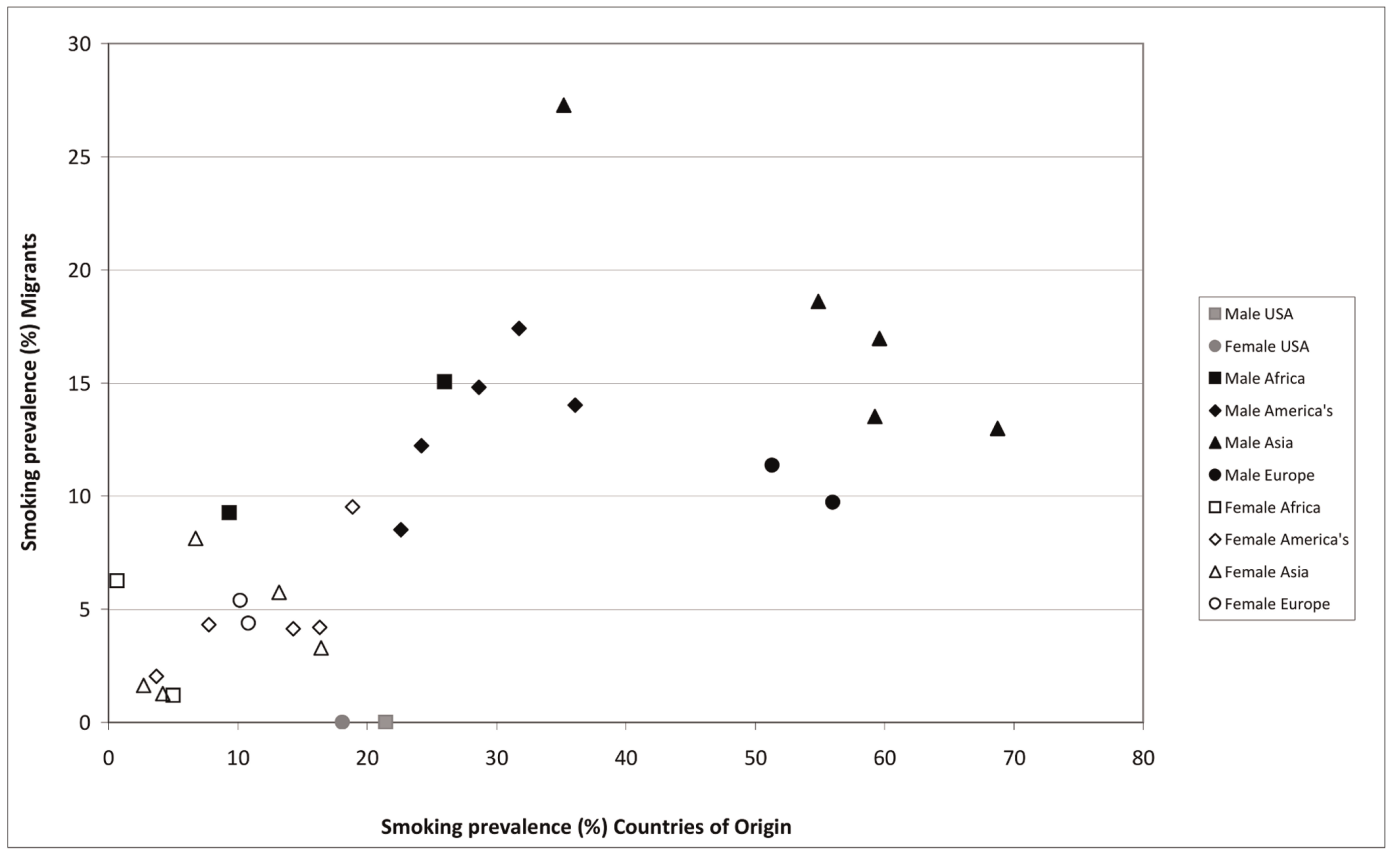
The difference in smoking prevalence between men and women (gender gap). *Other Africa: Ghana, Kenya and South Africa. **Total does not include US-born.

The gender gap (difference in smoking prevalence between men and women) among all migrant groups is smaller than in the countries of origin (figure 4). On the other hand, the gender gap among all migrants (except those from Ethiopia) is larger than for the US-born. Table 2 shows the relative risks of smoking for migrants compared to the US. RR’s for men are closer to 1.0 than those for women, indicating a smoking prevalence closer to the US-born population. Interactions between sex and country of origin are statistically significant for 8 out of 14 groups.

**Table 2:** The relative risk of smoking for migrants compared to the US-born group by country of origin^1^.

		RR (95% CI) Migrants/US			
		Total	Male	Female	P-value
**Africa**	**Ethiopia**	0.36 (0.15–0.84)	0.39 (0.15–1.02)	0.30 (0.06–1.60)	0.780
	**Other Africa** **	0.35 (0.20–0.62)	0.57 (0.32–1.00)	0.03 (0.00–0.19)	0.003*
**The Americas**	**Brazil**	0.63 (0.44–0.90)	0.73 (0.45–1.19)	0.54 (0.32–0.93)	0.415
	**Dom. Republic**	0.24 (0.17–0.35)	0.31 (0.19–0.50)	0.18 (0.10–0.33)	0.156
	**Ecuador**	0.58 (0.42–0.79)	0.78 (0.55–1.10)	0.25 (0.12–0.53)	0.007*
	**Guatemala**	0.45 (0.32–0.62)	0.61 (0.44–0.85)	0.11 (0.04–0.32)	0.003*
	**Mexico**	0.47 (0.44–0.50)	0.64 (0.59–0.69)	0.23 (0.20–0.26)	<0.001*
**Asia**	**China**	0.36 (0.29–0.46)	0.64 (0.51–0.81)	0.08 (0.04–0.16)	<0.001*
	**Laos**	0.78 (0.53–1.16)	1.09 (0.70–1.71)	0.47 (0.23–0.96)	0.051
	**Pakistan**	0.45 (0.26–0.79)	0.58 (0.32–1.06)	0.23 (0.06–0.92)	0.227
	**Philippines**	0.52 (0.44–0.61)	0.76 (0.63–0.92)	0.31 (0.24–0.42)	<0.001*
	**Vietnam**	0.44 (0.34–0.57)	0.77 (0.59–0.99)	0.08 (0.03–0.19)	<0.001*
**Europe**	**Russia**	0.32 (0.23–0.46)	0.46 (0.30–0.70)	0.20 (0.11–0.36)	0.026*
	**Ukraine**	0.45 (0.27–0.76)	0.52 (0.27–1.00)	0.38 (0.16–0.88)	0.556

1 Country of origin is country of birth, except for second generation migrants where it is the country of birth of the parents.

* Significant (p≤0.05).

** Other Africa: Ghana, Kenya and South Africa.

The relative risks of smoking for migrants stratified by their age at the time of entry to the US are shown in table 3. The expected linear pattern of lower RR’s with higher age at entry was only observed for migrants from the Philippines. P-values indicate that for almost all groups, statistically significant variation by age at entry exists. These variations however, do not follow a single pattern. For roughly half of the groups, RR’s are closest to 1.0 for the groups that were 40–85 years at entry. Only in four groups is the RR for the second generation migrants closest to 1.0.

**Table 3:** The relative risk of smoking for migrants compared to the US-born group stratified by age at migration to the US by country of origin^1^.

		RR Migrants/US (95% CI)				
		2nd gen.^2^	0–19	20–39	40–85	P-value^3^
**Africa**	**Ethiopia**	4	0.57 (0.15–2.11)	0.20 (0.06–0.72)	0.62 (0.10–3.90)	0.017*
	**Other Africa** **	4	0.52 (0.18–1.47)	0.31 (0.15–0.63)	0.65 (0.10–4.01)	<0.001*
**The Americas**	**Brazil**	4	0.19 (0.05–0.74)	0.66 (0.42–1.05)	1.81 (1.04–3.15)	<0.001*
	**Dom. Rep.**	0.32 (0.14–0.75)	0.15 (0.07–0.32)	0.28 (0.16–0.48)	0.27 (0.09–0.81)	<0.001*
	**Ecuador**	0.99 (0.49–1.97)	0.45 (0.24–0.86)	0.66 (0.44–1.01)	–	<0.001*
	**Guatemala**	0.23 (0.03–1.54)	0.35 (0.20–0.62)	0.54 (0.35–0.83)	0.60 (0.16–2.23)	<0.001*
	**Mexico**	0.40 (0.34–0.47)	0.44 (0.39–0.49)	0.52 (0.47–0.58)	0.60 (0.46–0.77)	<0.001*
**Asia**	**China**	0.25 (0.11–0.55)	0.23 (0.12–0.43)	0.50 (0.38–0.65)	0.22 (0.11–0.44)	<0.001*
	**Laos**	0.83 (0.40–1.73)	0.95 (0.55–1.64)	0.44 (0.16–1.25)	1.05 (0.30-3.62)	0.448
	**Pakistan**	0.70 (0.19–2.58)	0.48 (0.16–1.41)	0.35 (0.15–0.82)	0.53 (0.14–2.01)	0.018*
	**Philippines**	0.61 (0.42–0.88)	0.61 (0.46–0.81)	0.46 (0.36–0.59)	0.41 (0.25–0.68)	<0.001*
	**Vietnam**	0.16 (0.05–0.55)	0.52 (0.35-0.79)	0.56 (0.39–0.80)	0.27 (0.12–0.61)	<0.001*
**Europe**	**Russia**	0.14 (0.06–0.35)	0.47 (0.25–0.89)	0.55 (0.34–0.90)	0.11 (0.03–0.48)	<0.001*
	**Ukraine**	0.03 (0.00–0.21)	0.93 (0.44–1.95)	0.55 (0.25–1.22)	0.18 (0.04–0.75)	<0.001*

1 Country of origin is country of birth, except for second generation migrants where it is the country of birth of the parents.

2 2^nd^ Generation: those born in the US whose parents are both foreign-born.

3 Variance between all available categories.

4 No smokers in this group.

* Significant (p≤0.05).

** Other Africa: Ghana, Kenya and South Africa.

In table 4 the RR’s of smoking are displayed for each of the migrant groups stratified by the highest level of completed education. For all groups (Except Brazil, Laos and Vietnam) the RR of the highest education group was closer to 1.0 than the other groups, indicating a prevalence rate most similar to the US-born group of the same educational level. In five groups a linear pattern can be observed, where the higher educated groups have higher RR’s. Only in Laos a linear pattern is found in the opposite direction. Interaction between country of origin and education was statistically significant for all groups, except Laos.

**Table 4:** The relative risk of smoking for migrants with low, middle and high educational level compared to the US-born group of the same educational level, by country of origin^1^.

		RR Migrants/US (95%CI)			
		Low Education^2^	Middle Education^2^	High Education^2^	P-Value
**Africa**	**Ethiopia**	0.46 (0.08–2.77)	0.26 (0.09–0.75)	0.47 (0.09–2.61)	0.017*
	**Other Africa** **	0.01 (0.01–0.01)	0.27 (0.11–0.66)	0.75 (0.36–1.56)	<0.001*
**The Americas**	**Brazil**	1.09 (0.65–1.83)	0.39 (0.21–0.71)	0.76 (0.35–1.64)	<0.001*
	**Dom. Rep.**	0.26 (0.15–0.43)	0.11 (0.06–0.22)	0.54 (0.25–1.21)	<0.001*
	**Ecuador**	0.22 (0.10–0.51)	0.62 (0.42–0.93)	1.02 (0.54–1.93)	<0.001*
	**Guatemala**	0.40 (0.27–0.59)	0.27 (0.13–0.53)	0.87 (0.30–2.58)	<0.001*
	**Mexico**	0.39 (0.36–0.43)	0.38 (0.33–0.42)	0.58 (0.44–0.77)	<0.001*
**Asia**	**China**	0.30 (0.18–0.50)	0.38 (0.27–0.55)	0.52 (0.36–0.74)	<0.001*
	**Laos**	0.88 (0.53–1.46)	0.66 (0.37–1.19)	0.08 (0.01–0.58)	0.327
	**Pakistan**	0.48 (0.16–1.43)	0.24 (0.08–0.72)	0.88 (0.41–1.91)	0.006*
	**Philippines**	0.24 (0.14–0.40)	0.61 (0.48–0.76)	0.75 (0.59–0.95)	<0.001*
	**Vietnam**	0.30 (0.17–0.53)	0.57 (0.42–0.76)	0.26 (0.12-0.58)	<0.001*
**Europe**	**Russia**	0.09 (0.01–0.61)	0.24 (0.13–0.44)	0.70 (0.45–1.09)	<0.001*
	**Ukraine**	0.37 (0.07–1.96)	0.36 (0.14–0.92)	0.80 (0.41–1.57)	0.007*

1 Country of origin is country of birth, except for second generation migrants where it is the country of birth of the parents.

2 Education: Low = no higher than ‘12^th^ grade without diploma’, Middle = from ‘high school diploma or equivalent’ to ‘college but no degree’, High = from ‘associate degree’ to ‘doctorate degree’.

3 Group was too small.

* Significant (p≤0.05).

** Other Africa: Ghana, Kenya and South Africa.

## Discussion

The smoking prevalence among migrants was consistently lower than both the US-born group and the countries of origin. Our first hypothesis was not confirmed; patterns of variations in the prevalence of smoking between migrant groups were not very similar to those in the countries of origin. As hypothesized, the gender gap in smoking prevalence among all migrant groups (9.8%) was smaller than in the countries of origin (28.4%). Contrary to our expectations, generation and age at time of entry to the US were not systematically related to smoking prevalence for migrants. In accordance with our hypothesis, the risk of smoking for high-educated migrants was on average closer to their US counterparts.

### Limitations

Self-reported tobacco use is not the most reliable way to determine smoking status, as shown in a review containing studies from many countries, comparing self-reported smoking to cotinine measurements. [15] On average the prevalence estimated by self reports was 6% lower than by the cotinine measurement. Underestimation of tobacco use might have influenced our results if underestimation was much smaller or larger in specific migrant groups. One study on Southeast Asian immigrants found that self-reported cigarette use underestimated smoking prevalence compared to serum cotinine levels by 3% for men and 9% for women. [16] A study with a US-based Hispanic population reported differences in prevalence between self-report and cotinine measurements of 5.3% for men and 4.0% for women. [17] These studies suggest that underestimation of smoking prevalence among migrant groups is similar to that reported in the mentioned review. [15] Therefore, it seems that the risk of biased findings as a result of differential underestimation is small.

Although the WHS and TUS-CPS surveys measured smoking status in the same way, these rates might not be fully comparable. More detailed comparisons are prevented by the fact that in the WHS only current smoking status was asked for, while no information on former smoking or age of initiation was available. Apart from the surveying method (mostly telephone in the TUS-CPS and mostly household face-to-face interviewing in the WHS) there were no large differences between these surveys in sampling design or response rates and we made comparisons using the same standardized prevalence rates. Nonetheless, because of the large differences in context in which the two surveys were conducted, comparisons should be made with caution.

The timing of survey hampers an optimal comparison; the WHS was conducted in 2002–2003 and the TUS-CPS data were collected in 2006–2007. The interest for studying migrants mostly lies in comparing their current status to that in their country of origin at the time of migration. It is likely that the smoking prevalence in the country of origin has shifted through the stages of the tobacco epidemic since then. Because the WHS only provides data from 2002, any developments before or afterwards could not be investigated in this paper, but they have to be considered when interpreting these results.

### Interpretation of Results

The prevalence of smoking among migrants relative to other migrant groups does not seem to follow the patterns of their countries of origin relative to each other. The prevalence of smoking among Eastern European and especially Asian migrants is lower than expected given the relative rates of their countries of origin. The period of migration may be an important factor here. TUS-CPS data show that 50% of migrants from Asia came to the US before 1990 and from Eastern Europe before 1994. In contrast, most migrants from Africa came to the US after 1998 and from the America’s after 1994. The fact that Asian and Eastern European migrants have left their countries of origin long ago, and thus not experienced the local increases in smoking rates, might perhaps explain their lower smoking prevalence.

We found that the smoking prevalence among all migrant groups is much lower than in the countries of origin. This could be a result of exposure to US tobacco control policies, especially for the older migrant populations. In the US, like most developed countries, tobacco control policies have increased in comprehensiveness over recent years. [18] In many developing countries progress in tobacco control is slow, [19] and policies are undermined, for instance through smuggling to avoid taxes. [20] Due to large variations in the availability of data on this subject between the WHS countries, it is hard to make more detailed comparisons.

It has been argued that some tobacco control measures might have a limited effect on migrants in the US, due to differences in language and culture. [21], [22] On the other hand, a number of policies, such as tax/price increases and smoking bans do not necessarily rely on culture or language and therefore, in theory at least, may affect migrant groups as much as the majority population. Previous research has found no differences in the effect of tax increases and smoke-free policies between different racial/ethnic groups. [23] In addition, the price of cigarettes in the US is substantially higher than in the countries of origin. Both in absolute terms ($3.60 in the US vs. a mean of $0.92 in countries of origin) and adjusted for purchasing power parity ($3.71 in the US vs. a mean of $2.33 in countries of origin). [24] Combined with the knowledge that minority smokers are more likely to quit smoking as a result of price increases than the majority; [25] this might contribute to the relatively low smoking prevalence among migrants groups in the US.

Our finding that the prevalence of smoking among migrants is lower than that of the US-born is consistent with most studies comparing migrants to the host population.[26]–[28] The lower risk of smoking and smoking-related diseases for migrants has been explained by the ‘healthy migrant effect’. [6] This theory proposes that individuals with more socioeconomic resources, healthier life styles and better health may be more likely to migrate. While the effects of tobacco on physical health most often become apparent only at a later age, when the migrants are already settled in the US, many migrants will have started smoking before moving to the USA, based on the fact that 99% of adult smokers begin smoking before 26 years of age. [29] In our understanding, the healthy migrant effect includes not only physical health but also healthy behaviour. When smoking is taken as an example of healthy behaviour, our comparisons show that migrants are on average more healthy than the population that did not migrate, but further research is needed to corroborate this.

As we hypothesized, we found gender gaps in smoking among all migrant groups that were much smaller than in their countries of origin. However, this seems to have been the result of a different mechanism than expected. Generally the gender gap is thought to decrease as a result of an increase in women’s smoking. [30] However, as we found that female migrants smoke less than women in their countries of origin, the narrowing of the gender gap seems to be driven mostly by the low smoking prevalence among male migrants.

Age at migration did not show a consistent effect on smoking among migrants. The second generation migrants smoked less than the US-born population, as found in other studies. [6] In contrast to our expectations, however, they did not differ substantially from the first generation migrants. Moreover, the differences within the first generation, by time of immigration, do not support our expectations either. While these patterns are obviously in conflict with the theory of acculturation, [5] they could in some way be consistent with the theory of segmented assimilation. [7] This theory acknowledges that not all behaviours of migrants will be adapted to the host standards, but some behaviours will be retained from their country of origin. [8] Our results imply that the influence of the country of origin might be retained for a long time, even into the second generation.

### Conclusions

In this study we found that the smoking prevalence among migrants is consistently lower than both the country of origin levels and the US level. To understand these patterns, we tested four hypotheses that were derived from the theory of segmented assimilation. Of these four hypotheses, two were supported by our results, two were not. We conclude that, while the theory of segmented assimilation may be useful in understanding the smoking prevalence among migrants, elaborations based on further research are needed.

Other mechanisms that might help to understand the low smoking prevalence among migrants include the ‘healthy migrant effect’, according to which healthier (less smoking) people are more likely to migrate. Furthermore, it is important to take into account the stage of the smoking epidemic that the country of origin was in at the time of migration.

A better understanding of these mechanisms is important to predict future changes in smoking among migrants. For example, if the smoking prevalence in most developing countries will continue to increase as expected, [31] future migrants might not have the ‘privileged’ position that migrants today do have with regard to smoking. This could be prevented by implementation of tobacco control policies that have been found to be effective among migrants.
